# Gender disparity in access to advanced therapies for patients with Parkinson’s disease: a retrospective real-word study

**DOI:** 10.3389/fneur.2024.1429251

**Published:** 2024-09-25

**Authors:** Giuseppe Maccarrone, Gennaro Saporito, Patrizia Sucapane, Chiara Rizi, Federico Bruno, Alessia Catalucci, Maria Letizia Pistoia, Alessandra Splendiani, Alessandro Ricci, Ernesto Di Cesare, Marina Rizzo, Rocco Totaro, Francesca Pistoia

**Affiliations:** ^1^Department of Biotechnological and Applied Clinical Sciences, University of L’Aquila, L’Aquila, Italy; ^2^Department of Neurology, San Salvatore Hospital, L’Aquila, Italy; ^3^Department of Radiology, San Salvatore Hospital, L'Aquila, Italy; ^4^Department of Neurosurgery, San Salvatore Hospital, L’Aquila, Italy; ^5^Department of Neurology, Villa Sofia, Palermo, Italy

**Keywords:** Parkinson’s disease, MRgFUS, DBS, apomorphine, Duodopa

## Abstract

**Background:**

Gender differences in the access to advanced therapies for Parkinson’s disease (PD) are poorly investigated.

**Objective:**

The objective of this study was to investigate the presence of any gender disparity in the access to advanced therapies for PD.

**Design:**

Retrospective study.

**Methods:**

Data from patients with consistent access to the Parkinson’s and Movement Disorder Center of L’Aquila over the last 10-year period were screened. Patients selected for advanced therapies were included.

**Results:**

Out of 1,252 patients, 200 (mean age ± SD 71.02 ± 9.70; 72% males; median Hoen Yahr level: 3, minimum 1 maximum 5) were selected for advanced therapies: 133 for Magnetic Resonance guided Focused Ultrasound (MRgFUS) thalamotomy (mean age ± SD 70.0 ± 8.9; 77% males), 49 for Levodopa/Carbidopa Intestinal Gel (LCIG) infusion (mean age ± SD 74.3 ± 11.4; 59% males), 12 for Deep Brain Stimulation (DBS) (mean age ± SD 71.2 ± 6.3; 75% males), and 7 for Continuous Subcutaneous Apomorphine Infusion (CSAI) (mean age ± SD 69.7 ± 5.5; 43% males). No sex differences were found in relation to age (MRgFUS group: males vs. females 70.2 ± 8.9 vs. 70.8 ± 8.9, *p*-value = 0.809; LCIG group: males vs. females 73.5 ± 13.0 vs. 75.5 ± 8.5, *p*-value = 0.557; DBS group: males vs. females 77.2 ± 8.1 vs. 67.3 ± 8.6, *p*-value = 0.843; CSAI group: males vs. females 73.3 ± 4.0 vs. 67.0 ± 5.2, *p*-value = 0.144) and disease duration (MRgFUS group: males vs. females 8.3 ± 4.4 vs. 9.6 ± 6.7, *p*-value = 0.419; LCIG group: males vs. females 14.5 ± 5.81 vs. 17.3 ± 5.5; *p*-value = 0.205; DBS group: males vs. females 15.0 ± 9.6 vs. 15.5 ± 7.7, *p*-value = 0.796; CSAI group: males vs. females 11.7 ± 3.7 vs. 10.3 ± 3.7, *p*-value = 0.505).

**Conclusion:**

The predominance of males is higher than that expected based on the higher prevalence of PD in men. Women are less confident in selecting advanced therapies during the natural progression of their disease. Factors accounting for this discrepancy deserve further investigation.

## Introduction

Advanced therapies for PD include Levodopa/Carbidopa Intestinal Gel (LCIG) infusion, Continuous Subcutaneous Apomorphine Infusion (CSAI), Deep Brain Stimulation (DBS) and Magnetic Resonance guided Focused Ultrasound (MRgFUS) Thalamotomy (1–5). Recent international guidelines recommend the use of LCIG, apomorphine infusion and DBS for advanced PD with fluctuations not sufficiently managed with oral or transdermal treatments (6, 7). MRgFUS of the thalamus is recommended for medically resistant PD tremor within clinical studies or registries (6). While sex-related differences in epidemiological and clinical features of PD have been widely described, gender differences in the access to advanced therapies are poorly investigated. It is known that PD is more frequent in men than in women and that the age of disease onset is 2.1 years later in women as compared to men (8, 9). On the other hand, women usually take longer to seek treatment, encounter more problems during management and report a lower quality of life (8, 10). Moreover, women suffer from troublesome dyskinesias more frequently than men, due to different levodopa pharmacokinetics (8, 11). Women show a greater levodopa bioavailability than age-matched men, as evidenced by a higher area under the plasma concentration-time curve and a reduction in the oral drug clearance (11). This makes women particularly vulnerable to dyskinesias, especially in advanced stages of the disease when the clinical behavior mirrors the abrupt oscillations in plasma levodopa concentrations, thus leading to motor complications (wearing off, on–off, dyskinesias) (12).

Moving from this evidence, a greater request for advanced therapies in women might be expected. To date, no studies investigating this issue are available. Specific research on gender disparity utilization can provide valuable information on the different clinical course of PD among sexes and on the need of a gender-specific healthcare resource utilization in the complicated stages of the disease.

Hence, the aim of this study was to investigate the presence of any gender disparity in the access to LCIG, CSAI, DBS and MRgFUS in a large real-world sample of patients with advanced PD.

### Levodopa/Carbidopa Intestinal Gel Infusion

**Figure 1:**
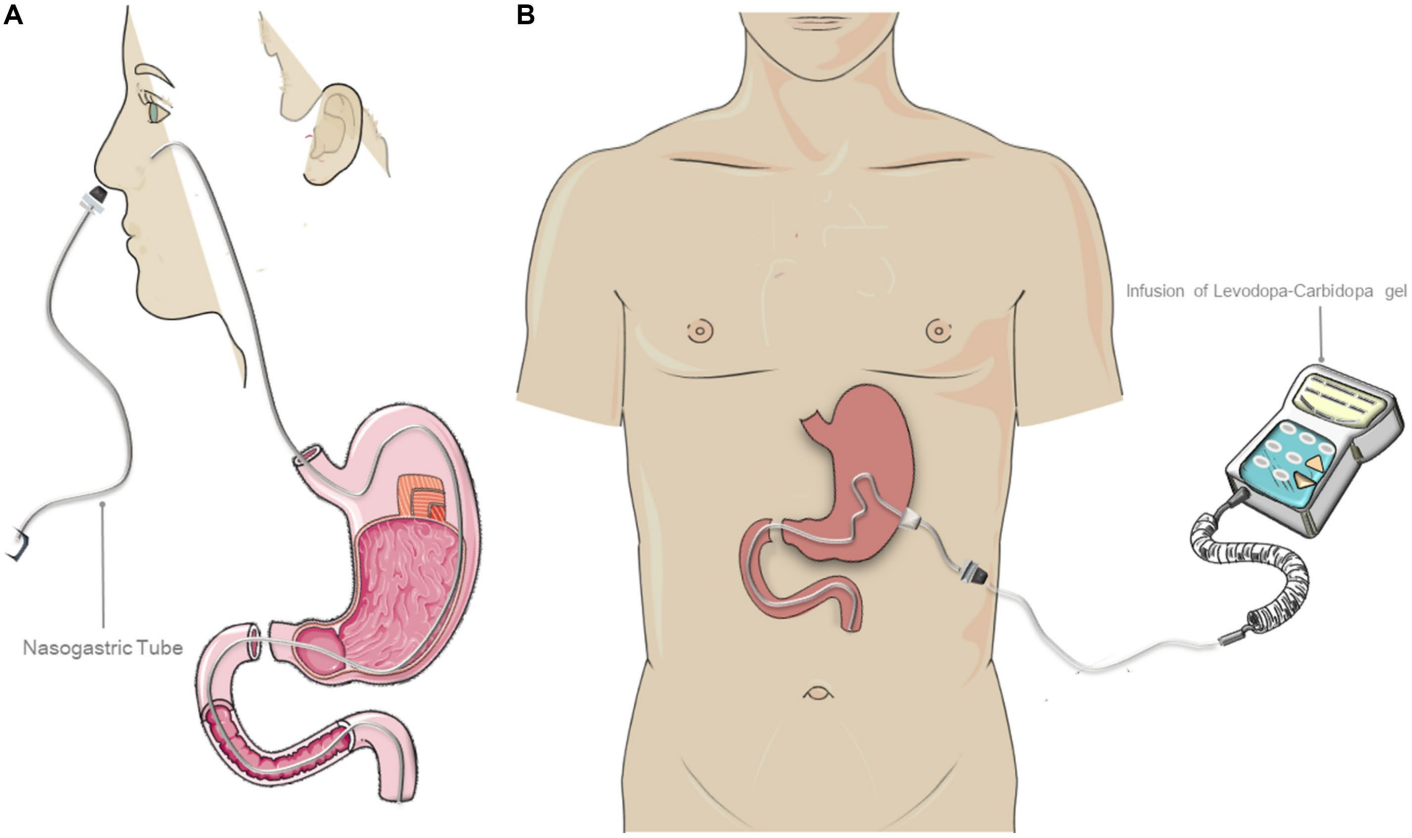
Treatment with levodopa-carbidopa intestinal gel (LCIG) infusion. **(A)** Representation of the “test” phase through temporary nasointestinal tube. This phase is performed to evaluate the treatment response. **(B)** Representation of the final Duodopa infusion system. The figure was partly generated using Servier Medical Art, provided by Servier, licensed under a Creative Commons Attribution 3.0 unported license.

LCIG is based on the use of a device that delivers a levodopa infusion into the proximal jejunum through a percutaneous endoscopic gastrostomy tube with a jejunal extension (PEG-J), connected to a portable infusion pump (Figure 1). It was developed with the aim of maintaining steady levels of levodopa in the bloodstream, by enhancing its absorption and minimizing the fluctuations that May occur over time with oral administration. Continuous administration reduces the likelihood of experiencing wearing-off periods, along with the associated motor and non-motor symptoms such as tremors, dystonia, bradykinesia, mood and sleep disturbances (13). Moreover, it reduces the likelihood of developing dyskinesia over time.

### Continuous Subcutaneous Apomorphine Infusion

**Figure 2:**
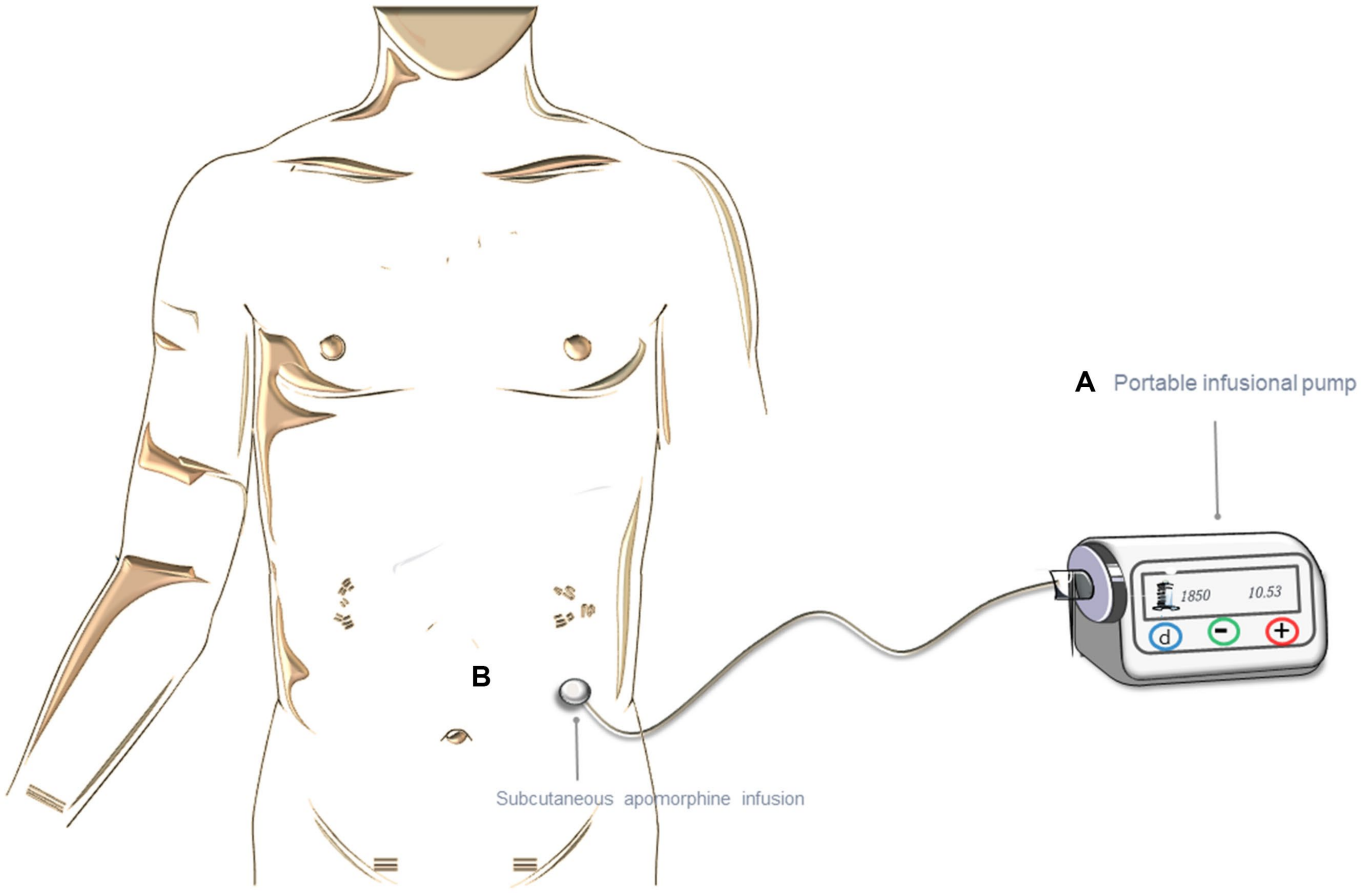
Treatment with continuous subcutaneous apomorphine infusion (CSAI). **(A)** Representation of the portable infusion pump. **(B)** Subcutaneous infusion. The figure was partly generated using Servier Medical Art, provided by Servier, licensed under a Creative Commons Attribution 3.0 unported license.

Apomorphine is a dopamine agonist, similar to L-dopa in terms of short half-life and D1/D2 receptor affinity (14, 15). It can be subcutaneously applied with a pen-injection or with a pump device for continuous delivery (Figure 2) (16). The dose of apomorphine is titrated while monitoring both the potential side effects and the effectiveness of the drug. Typically, the response is observed with a daily dose of apomorphine ranging from 3 to 30 mg through injection, or with an infusion rate of 1 to 4 mg per hour (equivalent to 0.1 mL to 0.4 mL) (1, 17). The infusion typically lasts around 12 to 16 h, with an interruption during the night, but, in some cases, it May be extended to 24 h.

### Deep Brain Simulation

**Figure 3:**
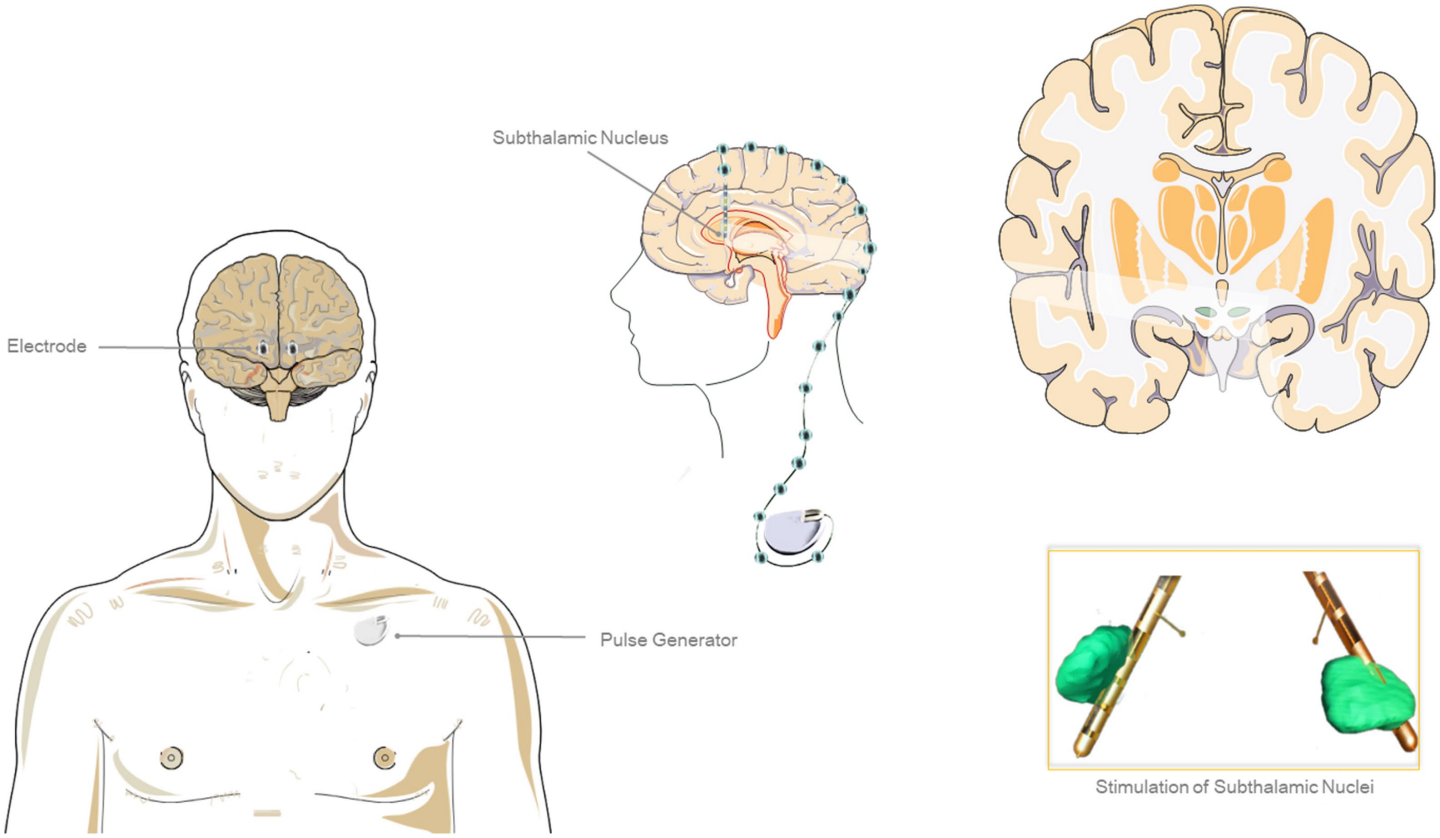
Deep brain stimulation (DBS) with subthalamic nuclei as targets. The figure was partly generated using Servier Medical Art, provided by Servier, licensed under a Creative Commons Attribution 3.0 unported license.

DBS is a surgical technique that involves the implantation of one or more electrodes, which are connected to a pulse generator delivering electrical stimuli into specific regions of the brain (18) (Figure 3). The potential targets include the subthalamic nucleus, the internal segment of the globus pallidus and the ventral-intermediate nucleus (VIM) of the thalamus (19). Microelectrodes are inserted through a hole in the skull, enabling the recording of neuronal signals to assess their proper positioning. During the surgical procedure, therapeutic stimulation can be delivered through the lead to evaluate responses in tremor, rigidity, and bradykinesia. Subsequently, the lead is anchored to the skull and the device is connected to a subcutaneous pocket with the impulse generator on the anterior chest wall. The start of stimulation is often delayed avoiding the lesion effect which could be a confounding factor for initial programming. However, the timings for the start of stimulation are not uniform and standardized across the different centers (5).

### Magnetic Resonance guided Focused Ultrasound Thalamotomy

**Figure 4:**
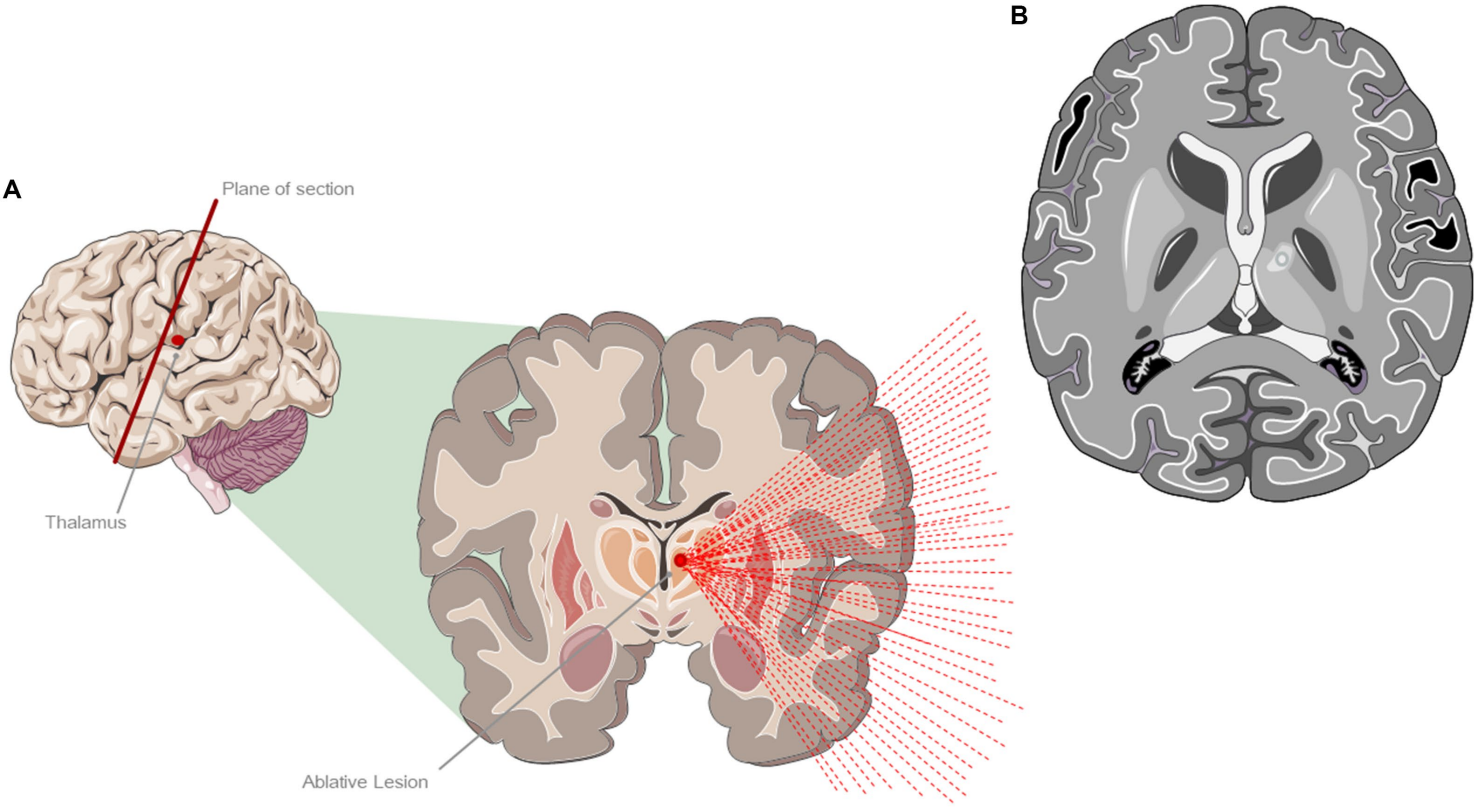
Treatment with magnetic resonance imaging-guided ultrasound (MRgFUS). **(A)** Ablative lesion of the left ventral intermediate (VIM) thalamic nucleus. **(B)** Representation of a characteristic lesion of the VIM at 24 h after the treatment. The figure shows the formation of a typical edema around the lesion area. The figure was partly generated using Servier Medical Art, provided by Servier, licensed under a Creative Commons Attribution 3.0 unported license.

This is an ablative approach that usually targets the thalamic ventral intermediate nucleus (Vim), which serves as a significant motor relay station involved in the development and maintenance of tremor (20) (Figure 4). Other targets are under investigation or in routine use in PD (Globus Pallidus, Subthalamic Nucleus, the Pallidothalamic Tract and the Cerebellothalamic Tract). It employs high-intensity focused ultrasound (HIFU), which, through the intact skull, generates hyperthermia (above 45°C), leading to coagulative tissue necrosis. Before the treatment begins a volumetric MRI sequence is acquired, which is merged with the previously obtained CT images to measure the skull density ratio (SDR). The focused ultrasound system is integrated into an MR scanner and the patient’s response is constantly monitored during the procedure. The day before the treatment, the patient undergoes complete shaving of the scalp and, on the day of the procedure, the stereotaxic frame is affixed to the skull. Sonications with gradually increasing energy and temperatures are performed (ranging from 46–52°C) to confirm the effectiveness of the treatment on the target area and assessing for any potential adverse effects. Once the target has been established the energy and temperature are escalated, reaching a maximum of 60°C causing an effective lesion (necrosis) with at least two sonications that have attained temperatures exceeding 56°C. As an important note, this procedure is the only one among the four considered that results in permanent and irreversible damage. Although the main indication for treatment is tremor, a general improvement of bradykinesia and rigidity has also been observed following the treatment (21). Frontal and executive functions, verbal fluency and memory, abstract reasoning and problem-solving abilities have been reported as completely preserved following the procedure (22).

## Methods

This was an observational retrospective real-word study. Data from patients with consistent access to the Parkinson’s and Movement Disorder Center of L’Aquila over the last 10-year period were collected and screened for the possible inclusion of patients in the study. Patients requiring advanced therapies were included in the study if referring to advanced therapies during the natural course of their disease. According to international guidelines, advanced therapy was defined as the treatment required when a patient consumed levodopa five times per day, experiences 2 hours of “off” symptoms, and 1 hour of troublesome dyskinesia within a day (named the “5-2-1” algorithm) (6, 23, 24). The selection among different advanced procedures was guided by the following criteria: indications for MRgFUS thalamotomy included the diagnosis of tremor-dominant PD, the lack of response to prior pharmacological treatments (Dopamine agonists, Carbidopa-Levodopa, Anticholinergic drugs), absence of contraindications for MRI (such as pacemaker, metal implants), and the detection of SDR values ≥0.35 (25, 26). Indications for DBS included a positive response to carbidopa-levodopa oral therapy, the absence of psychiatric disorders and cognitive impairment, the absence of major medical conditions preventing surgery, and the presence of a strong support network provided by relatives or caregivers. Indications for LCIG and CSAI included the satisfaction of the 5-2-1 criteria ascertained through medical history or the MANAGE-PD tool (27, 28). MANAGE-PD is a validated, web-based tool to assist physicians in identifying patients with PD whose symptoms are inadequately controlled by oral medications. For all included patients, anagraphical (age, sex) and main clinical data (disease type, disease duration, Hoen and Yahr scale stage) were collected. The Levodopa Equivalent Daily Dose (LEDD) that is a conversion factor derived from the total daily dosage of each PD medication taken by the patient, multiplied by a specific conversion factor unique to each medication was also estimated for all patients (29, 30). The mean SDR value was also considered for patients undergoing MRgFUS thalamotomy, as the SDR influences the permeability of the skull to the ultrasound waves and influences outcomes (25, 26). The study was approved by the Internal review Board of the University of L’Aquila (n. 18/2022).

### Statistical analysis

To assess potential gender differences among the mean values of the four groups, an independent samples *T*-test was performed. Continuous normally distributed variables (test variables) included age, disease duration, SDR and LEDD values while gender was the categorical variable with two categories (Grouping variables). To evaluate the role of other variables, an analysis of variance (ANOVA) was also performed including “severity of disease” (expressed by the Hoehn Yahr level) as a covariate and gender as fixed factor. To evaluate potential significant differences between the two groups, a Bonferroni post-hoc analysis was also conducted. Continuous variables were represented as mean ± standard deviation. Statistical significance was determined at an alpha level of 0.05. The analyses were performed using JAMOVI 2.2.24 software.

## Results

**Figure 5:**
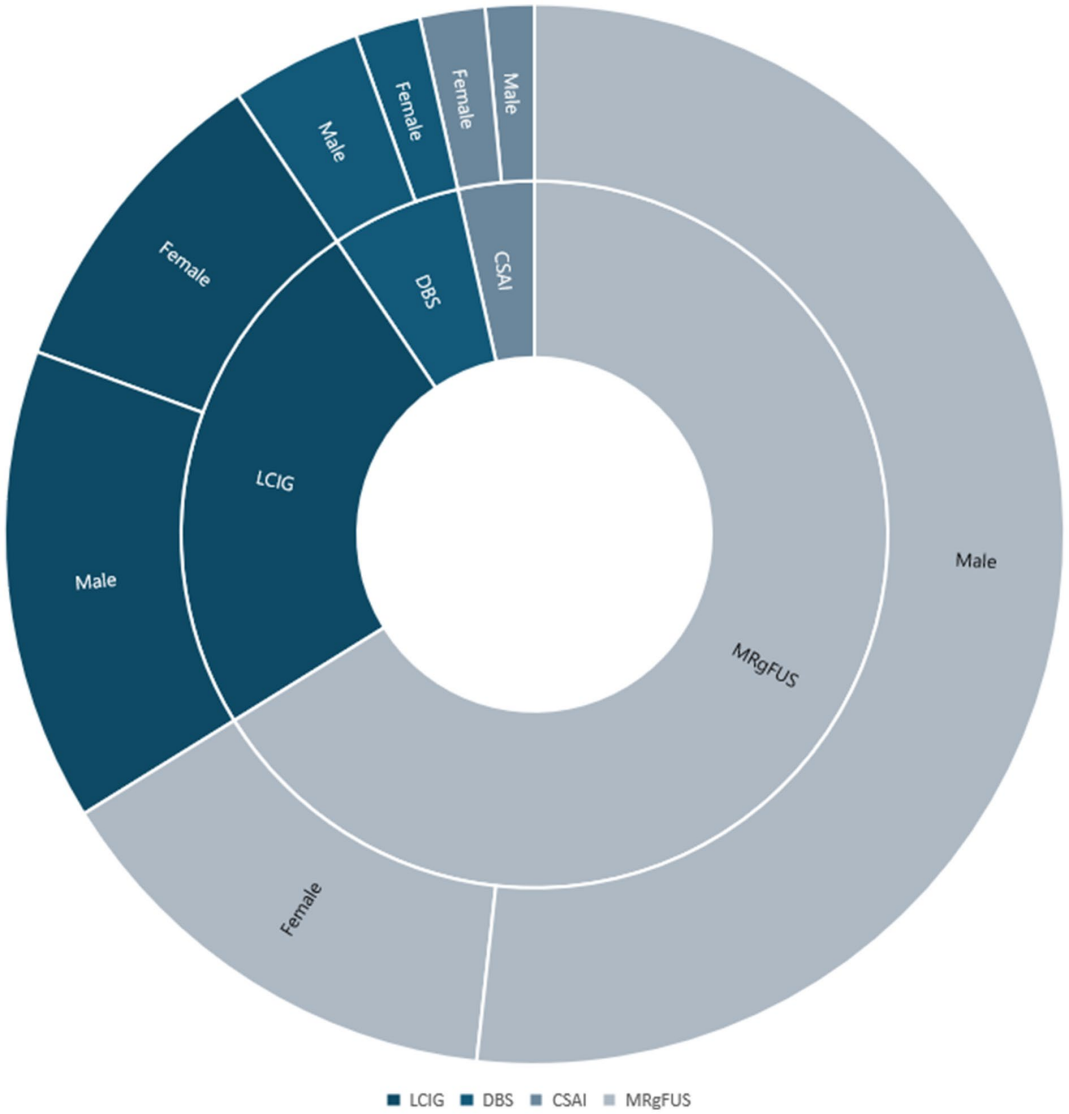
Gender disparities in access to advanced medical therapies. The present radial graph represents the distribution of men and women for different advanced therapies.

In the reference period 1,252 patients underwent regular periodic assessments at the Parkinson’s and Movement Disorder Center of L’Aquila (mean age ± SD 73.4 ± 9.9, 65% males). Out of them, 200 patients were considered eligible for advanced therapies (mean age ± SD 71.0 ± 9.7, 72% males, with a median Hoen and Yahr scale of 3, minimum 1 and maximum 5) and therefore included in the study. The most frequently performed advanced therapy was MRgFUS thalamotomy (*n* = 133; 68%, mean age ± SD 70.0 ± 8.9, 77% males), followed by LCIG (*n* = 49; 25%, mean age ± SD 74.3 ± 11.4, 59% males), DBS (*n* = 12; 6%: mean age ± SD 71.2 ± 6.3, 75% males) and CSAI (*n* = 7; mean age ± SD 69.70 ± 5.53; 43% males) (Figure 5).

**Figure 6:**
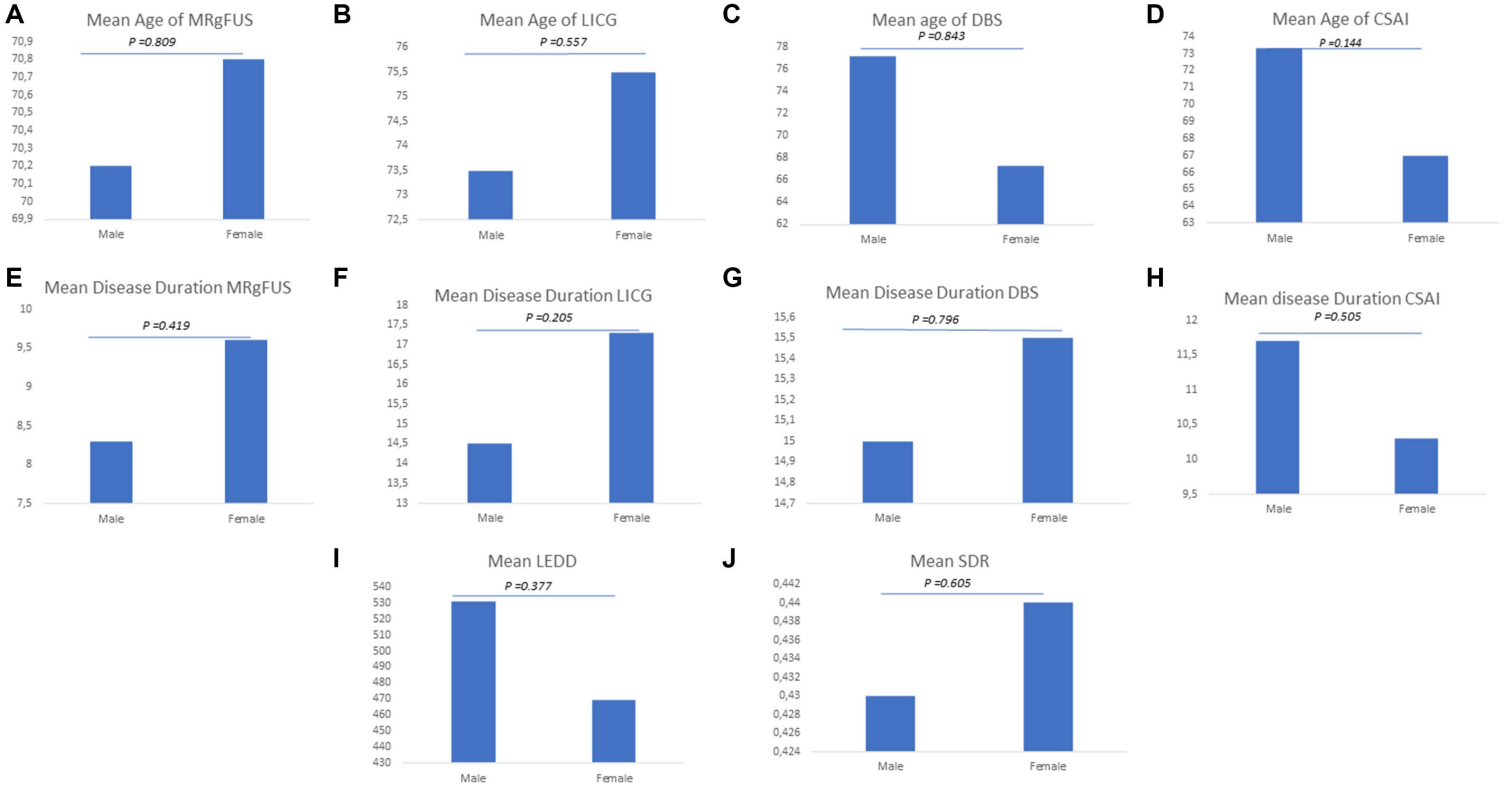
Explored clinical variables for advanced therapies. The figure graphically represents gender disparities considering the following clinic variables: **(A)** Mean age of MRgFUS; **(B)** Mean age of LICG; **(C)** Mean age of DBS; **(D)** Mean age of CSAI; **(E)** Mean disease of MRgFUS; **(F)** Mean disease of LICG; **(G)** Mean disease of DBS; **(H)** Mean disease of CSAI; **(I)** Mean LEDD; **(J)** Mean SDR.

As shown in Figures 6A–D, no sex differences were found in all groups in relation to age (MRgFUS group: males vs. females 70.20 ± 8.90 vs. 70.80 ± 8.90, *p*-value = 0.809; LCIG group: males vs. females 73.50 ± 13.20 vs. 75.50 ± 8.59, *p*-value = 0.557; DBS group: males vs. females 77.20 ± 8.10 vs. 67.30 ± 8.60, *p*-value = 0.843; CSAI group: males vs. females 73.30 ± 4.04 vs. 67.00 ± 5.23, *p*-value = 0.144) and disease duration (MRgFUS group: males vs. females 8.30 ± 4.40 vs. 9.60 ± 6.70, *p*-value = 0.419; LCIG group: males vs. females 14.50 ± 5.81 vs. 17.30 ± 5.53, *p*-value = 0.205; DBS group: males vs. females 15.00 ± 9.62 vs. 15.50 ± 7.78, *p*-value = 0.796; CSAI group: males vs. females 11.70 ± 3.79 vs. 10.30 ± 3.79, *p*-value = 0.505) (Figures 6E–H).

In the whole group, a higher mean LEDD was reported in men (mean LEDD±SD 531 ± 361 mg) than in women (mean LEDD±SD 469 ± 256 mg), although the difference was not statistically significant (*p*-value = 0.377). Moreover, in patients undergoing MRgFUS the mean SDR value was found to be slightly higher in women than in men (males vs. females 0.43 vs. 0.44), even though the difference was not statistically significant (*p*-value = 0.605) (Figures 6I,J).

**Figure 7:**
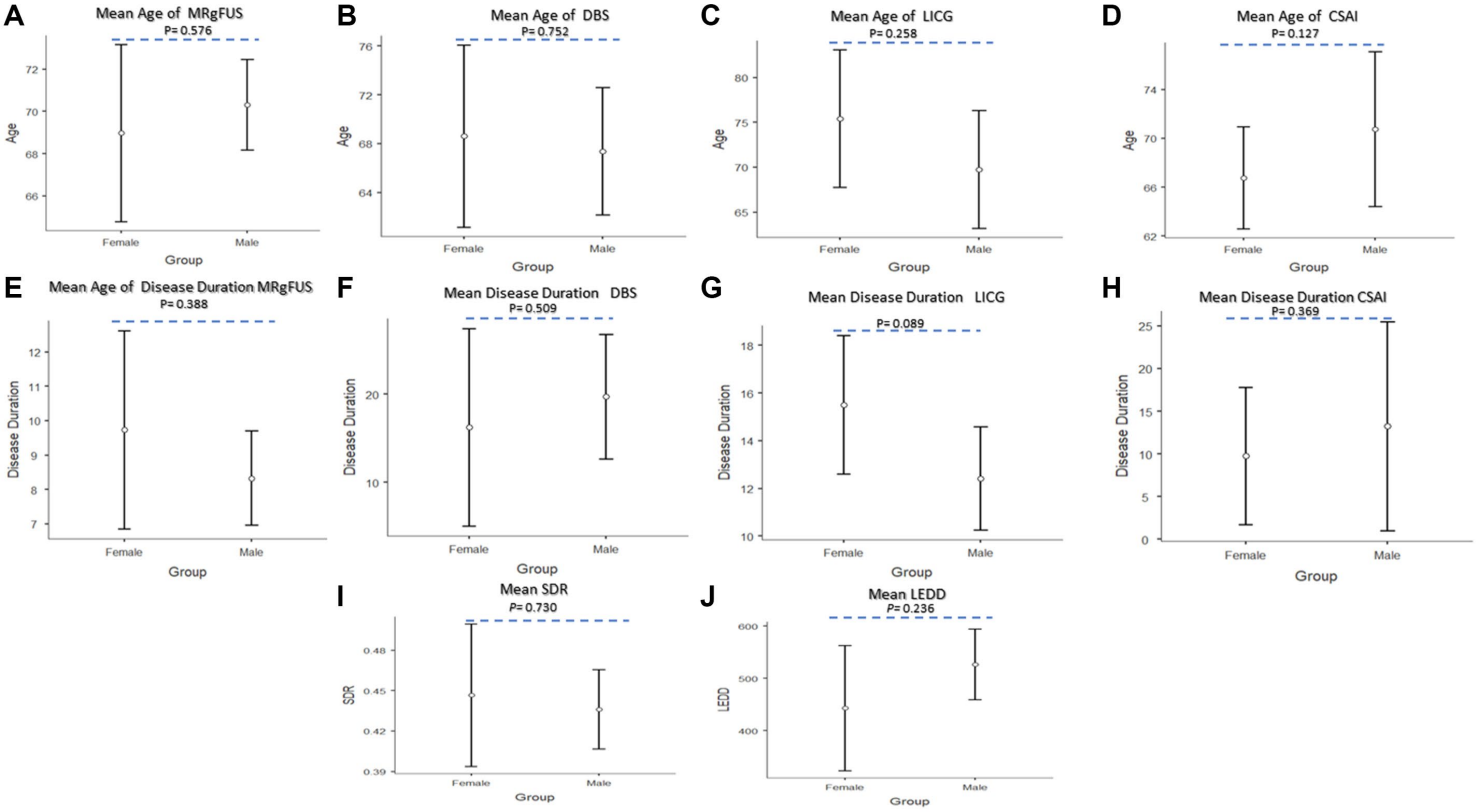
Post-hoc comparisons between the two groups (Female vs. Male) for advances therapies. **(A)** Mean age of MRgFUS; **(B)** Mean age of DBS; **(C)** Mean age of LICG; **(D)** Mean age of CSAI; **(E)** Mean disease duration MRgFUS; **(F)** Mean disease duration of DBS; **(G)** Mean disease duration LICG; **(H)** Mean disease duration CSAI; **(I)** Mean SDR; **(J)** Mean LEDD. The circle represent sample mean.

As reported in Figure 7, ANOVAs revealed no significant effect of the group factor on the test variables. The post-hoc comparisons (Figures 7A–H) revealed no significant differences in all groups in relation to age [MRgFUS (*p* = 0.567), DBS (*p* = 0.752), LICG (*p* = 0.258) and CSAI (*p* = 0.127)], and disease duration [MRgFUS (*p* = 0.388), DBS (*p* = 0.509), LICG (*p* = 0.089) and CSAI (*p* = 0.369)]. Moreover, post-hoc analyses showed that no statistically significant differences were observed in the whole sample concerning LEDD values (*p* = 0.236) and SDR values for patients undergoing MRgFUS (*p* = 0.730) (Figures 7I,J).

## Discussion

Our findings suggest the presence of a significant gender disparity in access to advanced therapies for PD. The observed gender discrepancy is greater than that expected based solely on the higher prevalence of PD in men. We considered all the clinical variables that might have driven this discrepancy, such as age, disease duration, the Hoehn Yahr level and the previous LEDD. None of these clinical variables appears to be distributed differently between the two genders, with small, recognized differences between sexes that are not statistically significant. With respect to MRgFUS, we hypothesized that women might have lower SDR values, potentially affecting their eligibility for the procedure. SDR is an indicator of sonication heating efficiency and sets patient-specific thresholds for the maximum temperature increase achievable with the technology (31). As noted in other MRgFUS studies, female patients tend to have significantly lower SDR and a larger skull volume compared to male patients (32). We hypothesized that the hormonal effects, which more frequently lead to osteoporosis in females, May have contributed to a lower SDR observed in female patients, potentially affecting their access to the procedure: this hypothesis is supported by earlier observations of a higher SDR in male patients compared to females undergoing MRgFUS (32). However, even this parameter was not distributed differently between the two sexes. This was in line with the previous research by Boutet et al., who examined a cohort of patients undergoing MRgFUS (33). They found no correlations between SDR, age, and sex, and concluded that SDR cannot be used as a criterion to explain the divergent access to MRgFUS thalamotomy between genders (33). To date, the available evidence regarding gender disparity in advanced therapies remains limited. Studies investigating gender disparity in the access to CSAI, LCIG, and MRgFUS are lacking, with only a few focusing on DBS. One of the pioneering studies investigating this issue revealed a predominantly male population in access to DBS without any gender-related differences observed in age at onset, disease progression rate, or disease severity (34). In a study by Somma et al., females showed more severe motor complications than men before undergoing DBS surgery, as well as a longer disease duration and a greater improvement than men after DBS surgery (35). Additional studies confirmed that women receive DBS at significantly lower rates than men in PD, although DBS has been reported as equally effective for both men and women (35–37). This finding seems to be attributed not only to differences in PD prevalence between the two sexes but also to other factors that account for disparate access (37–41). Shpiner et al. found that a significant proportion of women did not undergo surgery simply due to patient preferences (42).

A similar pattern was noted by Jost et al., who observed that among patients selected for DBS, the proportion of women who ultimately underwent DBS surgery was smaller due to the patients’ desire for additional time for reflection or a preference for further medical optimization, despite the expected equal post-surgery efficacy for both sexes (43). This occurred despite the fact that more women than men were initially selected for DBS surgery, partly because they had a more severe disease status and worse quality of life (e.g., more severe motor fluctuations) before the evaluation. According to the authors, a greater fear of surgery and a higher prevalence of depression in women might have contributed to the final pattern of access (43). Similarly, Hendriks et al. found that women had a higher likelihood of being candidate for DBS, but a lower proportion ultimately underwent DBS surgery, due to personal preferences or mood disorders (44). Interestingly, the authors highlighted that while men tended to have a decision-making process driven by their own initiative, women tended to hesitate and wait, being more anxious and appearing more fearful about complications. Women seemed to require more support from their social environment and ultimately refused surgery at a higher rate (44). Furthermore, women who underwent surgery exhibited a notable increase in impulsive behavior, which could signify an improvement in their hesitant attitude before surgery (45). Additionally, after surgery, both men and women experienced an increase in body mass index, but men gained both lean and fat mass, whereas women gained only fat mass (44). Other studies confirmed a lower access to DBS for women and attributed this difference either to differences in confidence in undergoing surgery (46, 47) or to financial limitations (48). Another aspect of the discussion has been highlighted by the study of Sarica et al., who demonstrated that while females had significantly lower chances of undergoing DBS implantation as compared to males, this difference was more pronounced in patients with PD as compared to patients with other movement disorders (49). One possible explanation could be that female PD patients tend to show more psychiatric or psychological comorbidities, such as depression or a lower tendency to make decisions, compared to male PD patients (49). In this context, Hamberg et al. indeed demonstrated that women were less likely to take their own initiative in pursuing surgery, often expressing a strong fear of complications. They were also more inclined to consult friends and relatives before deciding to undergo DBS surgery (50). As a result, women with PD appear to be less likely to be referred for DBS evaluation by general practitioners and neurologists, especially for personal reasons and preferences (45, 50).

Taken together, this evidence suggests that the differential access between genders to advanced therapies could be explained by a distinct psychological and decision-making substrate, which significantly influences whether individuals opt to pursue advanced therapies or not. Peculiar decision-making styles together with a tendency to apathy has been described in PD patients (51). Moreover, studies investigating the different personalities of PD patients showed that women were more inclined towards hypochondria, depression, functional neurological disorders, and social isolation (52–54). Since depression, and consequently apathy, tend to be more prevalent in women with PD, this might help to explain why female PD patients are less inclined to seek out advanced PD therapies. Moreover, women with PD have a lower access to caregiving as compared to men, despite the finding that caregivers of women report less strain than those of men (55). In addition, women are more likely to use formal, paid caregivers (55). This May further increase the tendency of women to postpone access to advanced therapies, opting instead for less invasive options in the meantime. The observation, in our study, that CSAI is the least invasive therapy offered, and the only advanced therapy showing no significant gender discrepancy, is consistent with this interpretation. CSAI is the only technique that does not require shaving (unlike DBS or MRgFUS), or the setup of an invasive drug-delivery device (as in the case of LCIG). This makes it appear as a safer and more sustainable option for women, even with fewer aesthetic repercussions. However, differences in psychological and decision-making styles between genders, as potential reasons for disparity, were not investigated in this study. This is because their role was not hypothesized *a priori* but it was considered only after ruling out the influence of other specifically investigated clinical variables such as age, disease duration, the Hoehn Yahr level and the previous LEDD. Future studies should specifically investigate psychological factors and social traits using standardized assessments and interviews to confirm this hypothesis.

Strengths of our study include the novelty of findings, as it is the first study exploring gender disparity with respect to the whole spectrum of available advanced therapies (DBS, CSAI, MRgFUS, LCIG), and the large sample investigated. The limitations of the study May include the retrospective nature of the study design and the fact that not all subgroups of advanced therapies were equally represented numerically. Another limitation is that the research was conducted at a single center, which means the results May be influenced by the experience and preferences of the providers. Consequently, the conclusions May not be generalizable. Future multicenter cohort studies examining the entire timeline of patient management, from selection to treatment, could help determine whether there is a gender disparity in the interval from PD diagnosis to treatment for MRgFUS treatments as well for other advanced therapies. Moreover, as previously discussed, future studies should consider and investigate the role of psychological factors and social traits as potential contributors to gender disparity. In conclusion, we can state that the differential access to advanced therapies between sexes cannot be solely explained by the higher prevalence of PD in men, nor by the distribution of the different clinical variables investigated, which were not differently distributed among sexes. Thus, gender differences May be attributed to psychological, behavioral, or even aesthetic issues, as well as different decision-making styles between females and males. Altogether, these factors warrant further investigation, especially considering the impact that gender disparities can have in clinical practice. The potential consequences of gender disparities in access to advanced PD therapies include different outcomes based on gender. Especially if it is confirmed that differences in access to advanced therapies for PD are linked to psychological, social, and cultural factors, it will be necessary to raise awareness to try to mitigate those factors that currently negatively affect women’s access to advanced care. Specific interventions and educational initiatives could be implemented to address the observed differences and design comprehensive care tailored to the specific needs of patients, including considerations based on gender. Further studies need to be performed to further explore the reasons behind this disparity and to encourage female PD patients to seek advanced therapies that can improve their symptoms and quality of life.

## Data Availability

The raw data supporting the conclusions of this article will be made available by the authors, without undue reservation.

## References

[1] Müller T. An evaluation of subcutaneous apomorphine for the treatment of Parkinson's disease. Expert Opin Pharmacother. (2020) 21:1659–65. doi: 10.1080/14656566.2020.1787379 32640853

[2] Bruno F Catalucci A Arrigoni F Sucapane P Cerone D Cerrone P. An experience-based review of HIFU in functional interventional neuroradiology: transcranial MRgFUS thalamotomy for treatment of tremor. Radiol Med. (2020) 125:877–86. doi: 10.1007/s11547-020-01186-y, PMID: 32266693

[3] Thakkar S Fung VSC Merola A Rollins M Soileau MJ Kovács N. 24-hour levodopa-carbidopa intestinal gel: clinical experience and practical recommendations. CNS Drugs. (2021) 35:137–49. doi: 10.1007/s40263-020-00782-w, PMID: 33582982 PMC7907013

[4] Wirdefeldt K Odin P Nyholm D. Levodopa-carbidopa intestinal gel in patients with Parkinson's disease: a systematic review. CNS Drugs. (2016) 30:381–404. doi: 10.1007/s40263-016-0336-5 27138916

[5] Okun MS. Deep-brain stimulation for Parkinson's disease. N Engl J Med. (2012) 367:1529–38. doi: 10.1056/NEJMct1208070 23075179

[6] Deuschl G Antonini A Costa J Śmiłowska K Berg D Corvol JC. European academy of neurology/Movement Disorder Society-European section guideline on the treatment of Parkinson's disease: I. Invasive therapies. Mov Disord. (2022) 37:1360–74. doi: 10.1002/mds.29066, PMID: 35791767

[7] Abusrair AH Elsekaily W Bohlega S. Tremor in Parkinson's disease: from pathophysiology to advanced therapies. Tremor Other Hyperkinet Mov (N Y). (2022) 12:29. doi: 10.5334/tohm.712, PMID: 36211804 PMC9504742

[8] Crispino P Gino M Barbagelata E Ciarambino T Politi C Ambrosino I. Gender differences and quality of life in Parkinson's disease. Int J Environ Res Public Health. (2020) 18:198. doi: 10.3390/ijerph18010198, PMID: 33383855 PMC7795924

[9] Baba Y Putzke JD Whaley NR Wszolek ZK Uitti RJ. Gender and the Parkinson's disease phenotype. J Neurol. (2005) 252:1201–5. doi: 10.1007/s00415-005-0835-7 16151602

[10] Patel R Kompoliti K. Sex and gender differences in Parkinson's disease. Neurol Clin. (2023) 41:371–9. doi: 10.1016/j.ncl.2022.12.001 37030964

[11] Contin M Lopane G Belotti LMB Galletti M Cortelli P Calandra-Buonaura G. Sex is the Main determinant of levodopa clinical pharmacokinetics: evidence from a large series of levodopa therapeutic monitoring. J Parkinsons Dis. (2022) 12:2519–30. doi: 10.3233/JPD-223374, PMID: 36373294 PMC9837688

[12] Armstrong MJ Okun MS. Diagnosis and treatment of Parkinson disease: a review. JAMA. (2020) 323:548–60. doi: 10.1001/jama.2019.22360 32044947

[13] Nutt JG. Pharmacokinetics and pharmacodynamics of levodopa. Mov Disord. (2008) 23:S580–4. doi: 10.1002/mds.22037 18781675

[14] Wagner MJ Daniel CP Plaisance CJ Borne GE Ahmadzadeh S Shekoohi S. Apomorphine for Parkinson's disease: pharmacologic and clinical considerations. Expert Opin Emerg Drugs. (2023) 28:275–81. doi: 10.1080/14728214.2023.2278677, PMID: 37909462

[15] Deleu D Hanssens Y Northway MG. Subcutaneous apomorphine: an evidence-based review of its use in Parkinson's disease. Drugs Aging. (2004) 21:687–709. doi: 10.2165/00002512-200421110-00001 15323576

[16] Wenzel K Homann CN Fabbrini G Colosimo C. The role of subcutaneous infusion of apomorphine in Parkinson's disease. Expert Rev Neurother. (2014) 14:833–43. doi: 10.1586/14737175.2014.928202, PMID: 24917215

[17] Frankel JP Lees AJ Kempster PA Stern GM. Subcutaneous apomorphine in the treatment of Parkinson's disease. J Neurol Neurosurg Psychiatry. (1990) 53:96–101. doi: 10.1136/jnnp.53.2.96, PMID: 2313313 PMC487943

[18] Okun MS. Deep-brain stimulation--entering the era of human neural-network modulation. N Engl J Med. (2014) 371:1369–73. doi: 10.1056/NEJMp1408779 25197963

[19] Bronstein JM Tagliati M Alterman RL Lozano AM Volkmann J Stefani A. Deep brain stimulation for Parkinson disease: an expert consensus and review of key issues. Arch Neurol. (2011) 68:165. doi: 10.1001/archneurol.2010.260, PMID: 20937936 PMC4523130

[20] Taranta V Saporito G Ornello R Splendiani A Bruno F Sucapane P. Magnetic resonance-guided focused ultrasound thalamotomy for refractory neuropathic pain: a systematic review and critical appraisal of current knowledge. Ther Adv Neurol Disord. (2023) 16:17562864231180729. doi: 10.1177/17562864231180729, PMID: 37363184 PMC10286169

[21] Yin C Zong R Song G Zhou J Pan L Li X. Comparison of motor scores between OFF and ON states in tremor-dominant Parkinson's disease after MRgFUS treatment. J Clin Med. (2022) 11:4502. doi: 10.3390/jcm11154502, PMID: 35956119 PMC9369361

[22] Saporito G Sucapane P Ornello R Cerone D Bruno F Splendiani A. Cognitive outcomes after focused ultrasound thalamotomy for tremor: results from the COGNIFUS (COGNitive in focused UltraSound) study. Parkinsonism Relat Disord. (2023) 106:105230. doi: 10.1016/j.parkreldis.2022.105230, PMID: 36470172

[23] Brinker D Smilowska K Paschen S Antonini A Moro E Deuschl G. How to use the new European academy of neurology/Movement Disorder Society European section guideline for invasive therapies in Parkinson's disease. Mov Disord Clin Pract. (2024) 11:209–19. doi: 10.1002/mdc3.13962, PMID: 38214401 PMC10928336

[24] Rodríguez-Martín D Cabestany J Pérez-López C Pie M Calvet J Samà A. A new paradigm in Parkinson's disease evaluation with wearable medical devices: a review of STAT-ON™. Front Neurol. (2022) 13:912343. doi: 10.3389/fneur.2022.912343, PMID: 35720090 PMC9202426

[25] Ghanouni P Pauly KB Elias WJ Henderson J Sheehan J Monteith S. Transcranial MRI-guided focused ultrasound: a review of the technologic and neurologic applications. AJR Am J Roentgenol. (2015) 205:150–9. doi: 10.2214/AJR.14.13632, PMID: 26102394 PMC4687492

[26] Chang WS Jung HH Zadicario E Rachmilevitch I Tlusty T Vitek S. Factors associated with successful magnetic resonance-guided focused ultrasound treatment: efficiency of acoustic energy delivery through the skull. J Neurosurg. (2016) 124:411–6. doi: 10.3171/2015.3.JNS142592, PMID: 26361280

[27] Antonini A Stoessl AJ Kleinman LS Skalicky AM Marshall TS Sail KR. Developing consensus among movement disorder specialists on clinical indicators for identification and management of advanced Parkinson’s disease: a multi-country Delphi-panel approach. Curr Med Res Opin. (2018) 34:2063–73. doi: 10.1080/03007995.2018.1502165 30016901

[28] Antonini A Odin P Schmidt P Cubillos F Standaert DG Henriksen T. Validation and clinical value of the MANAGE-PD tool: a clinician-reported tool to identify Parkinson's disease patients inadequately controlled on oral medications. Parkinsonism Relat Disord. (2021) 92:59–66. doi: 10.1016/j.parkreldis.2021.10.009, PMID: 34695657

[29] Julien C Hache G Dulac M Dubrou C Castelnovo G Giordana C. The clinical meaning of levodopa equivalent daily dose in Parkinson's disease. Fundam Clin Pharmacol. (2021) 35:620–30. doi: 10.1111/fcp.12646, PMID: 33458868

[30] Tomlinson CL Stowe R Patel S Rick C Gray R Clarke CE. Systematic review of levodopa dose equivalency reporting in Parkinson's disease. Mov Disord. (2010) 25:2649–53. doi: 10.1002/mds.23429, PMID: 21069833

[31] Yang AI Hitti FL Alabi OO Joshi D Chaibainou H Henry L. Patient-specific effects on sonication heating efficiency during magnetic resonance-guided focused ultrasound thalamotomy. Med Phys. (2021) 48:6588–96. doi: 10.1002/mp.15239, PMID: 34532858

[32] Chang KW Park YS Chang JW. Skull factors affecting outcomes of magnetic resonance-guided focused ultrasound for patients with essential tremor. Yonsei Med J. (2019) 60:768–73. doi: 10.3349/ymj.2019.60.8.768, PMID: 31347332 PMC6660436

[33] Boutet A Gwun D Gramer R Ranjan M Elias GJB Tilden D. The relevance of skull density ratio in selecting candidates for transcranial MR-guided focused ultrasound. J Neurosurg. (2019) 132:1785–91. doi: 10.3171/2019.2.JNS182571, PMID: 31051458

[34] Accolla E Caputo E Cogiamanian F Tamma F Mrakic-Sposta S Marceglia S. Gender differences in patients with Parkinson's disease treated with subthalamic deep brain stimulation. Mov Disord. (2007) 22:1150–6. doi: 10.1002/mds.21520 17469208

[35] Somma T Bove I Vitulli F Solari D Bocchino A Palmiero C. Gender gap in deep brain stimulation for Parkinson's disease: preliminary results of a retrospective study. Neurosurg Rev. (2024) 47:63. doi: 10.1007/s10143-024-02290-7, PMID: 38263479 PMC10806036

[36] Chiou SM. Sex-related prognostic predictors for Parkinson disease undergoing subthalamic stimulation. World Neurosurg. (2015) 84:906–12. doi: 10.1016/j.wneu.2015.05.023, PMID: 26038335

[37] Venkatraman V Futch BG Bode Padron KJ Yang LZ Lee HJ Seas A. Disparities in the treatment of movement disorders using deep brain stimulation. J Neurosurg. (2024) 141:1–11. doi: 10.3171/2023.11.JNS23882, PMID: 38306639 PMC10898494

[38] Dalrymple WA Pusso A Sperling SA Flanigan JL Huss DS Harrison MB. Comparison of Parkinson’s disease patients’ characteristics by indication for deep brain stimulation: men are more likely to have DBS for tremor. Tremor Other Hyperkinet Mov (N Y). (2019) 9. doi: 10.7916/tohm.v0.676 PMC6749750 31572622

[39] Golfrè Andreasi N Romito LM Telese R Cilia R Elia AE Novelli A. Short-and long-term motor outcome of STN-DBS in Parkinson's disease: focus on sex differences. Neurol Sci. (2022) 43:1769–81. doi: 10.1007/s10072-021-05564-w, PMID: 34499244

[40] Blomstedt P Sandvik U Hariz MI Fytagoridis A Forsgren L Hariz GM. Influence of age, gender and severity of tremor on outcome after thalamic and subthalamic DBS for essential tremor. Parkinsonism Relat Disord. (2011) 17:617–20. doi: 10.1016/j.parkreldis.2011.05.014, PMID: 21676643

[41] Kübler D Astalosch M Gaus V Krause P de Almeida Marcelino AL Schneider GH. Gender-specific outcomes of deep brain stimulation for Parkinson's disease – results from a single movement disorder center. Neurol Sci. (2023) 44:1625–31. doi: 10.1007/s10072-023-06598-y, PMID: 36607479 PMC10102088

[42] Shpiner DS Di Luca DG Cajigas I Diaz JS Margolesky J Moore H. Gender disparities in deep brain stimulation for Parkinson's disease. Neuromodulation. (2019) 22:484–8. doi: 10.1111/ner.12973, PMID: 31120180

[43] Jost ST Strobel L Rizos A Loehrer PA Ashkan K Evans J. EUROPAR and the international Parkinson and movement disorders society non-motor Parkinson’s disease study group. Gender gap in deep brain stimulation for Parkinson's disease. NPJ Parkinsons Dis. (2022) 8:47. doi: 10.1038/s41531-022-00305-y, PMID: 35444187 PMC9021281

[44] Hendriks M Vinke RS Georgiev D. Gender discrepancies and differences in motor and non-motor symptoms, cognition, and psychological outcomes in the treatment of Parkinson's disease with subthalamic deep brain stimulation. Front Neurol. (2024) 14:1257781. doi: 10.3389/fneur.2023.1257781, PMID: 38259647 PMC10800523

[45] Memon AA Gelman K Melott J Billings R Fullard M Catiul C. A systematic review of health disparities research in deep brain stimulation surgery for Parkinson's disease. Front Hum Neurosci. (2023) 17:1269401. doi: 10.3389/fnhum.2023.1269401, PMID: 37964803 PMC10641459

[46] Reker P Jost ST Schiller P Gronostay A Fink GR Visser-Vandewalle V. Gender differences in referrals for deep brain stimulation for essential tremor. Parkinsonism Relat Disord. (2023) 112:105490. doi: 10.1016/j.parkreldis.2023.105490 37354776

[47] Watanabe G Morden FTC Gao F Morita M Bruno MK. Utilization and gender disparities of deep brain stimulation surgery amongst Asian Americans, native Hawaiians, and other Pacific islanders with Parkinson's disease in Hawai’i. Clin Neurol Neurosurg. (2022) 222:107466. doi: 10.1016/j.clineuro.2022.107466, PMID: 36209519

[48] Chandran S Krishnan S Rao RM Sarma SG Sarma PS Kishore A. Gender influence on selection and outcome of deep brain stimulation for Parkinson's disease. Ann Indian Acad Neurol. (2014) 17:66–70. doi: 10.4103/0972-2327.128557, PMID: 24753663 PMC3992773

[49] Sarica C Conner CR Yamamoto K Yang A Germann J Lannon MM. Trends and disparities in deep brain stimulation utilization in the United States: a Nationwide inpatient sample analysis from 1993 to 2017. Lancet Reg Health Am. (2023) 26:100599. doi: 10.1016/j.lana.2023.100599, PMID: 37876670 PMC10593574

[50] Hamberg K Hariz GM. The decision-making process leading to deep brain stimulation in men and women with Parkinson’s disease—an interview study. BMC Neurol. (2014) 14:89. doi: 10.1186/1471-2377-14-89 24761767 PMC4000892

[51] Gilmour W Mackenzie G Feile M Tayler-Grint L Suveges S Macfarlane JA. Impaired value-based decision-making in Parkinson's disease apathy. Brain. (2024) 147:1362–76. doi: 10.1093/brain/awae025, PMID: 38305691 PMC10994558

[52] Santangelo G Piscopo F Barone P Vitale C. Personality in Parkinson's disease: clinical, behavioural and cognitive correlates. J Neurol Sci. (2017) 374:17–25. doi: 10.1016/j.jns.2017.01.013 28087060

[53] Perrin AJ Nosova E Co K Book A Iu O Silva V. Gender differences in Parkinson's disease depression. Parkinsonism Relat Disord. (2017) 36:93–7. doi: 10.1016/j.parkreldis.2016.12.026 28089265

[54] Colautti L Iannello P Silveri MC Antonietti A. Decision-making under ambiguity and risk and executive functions in Parkinson's disease patients: a scoping review of the studies investigating the Iowa gambling task and the game of dice. Cogn Affect Behav Neurosci. (2023) 23:1225–43. doi: 10.3758/s13415-023-01106-3, PMID: 37198383 PMC10545597

[55] Dahodwala N Shah K He Y Wu SS Schmidt P Cubillos F. Sex disparities in access to caregiving in Parkinson disease. Neurology. (2018) 90:e48–54. doi: 10.1212/WNL.0000000000004764, PMID: 29196580 PMC10681055

